# Protein Intake from Various Foods Sources Is Negatively Associated with Cardiometabolic Risk Markers in Italian Older Adults

**DOI:** 10.1007/s12603-023-1981-2

**Published:** 2023-09-30

**Authors:** Hélio José Coelho-Júnior, R. Calvani, A. Picca, G. Savera, M. Tosato, F. Landi, Emanuele Marzetti

**Affiliations:** 1 Department of Geriatrics, Orthopedics and Rheumatology, Università Cattolica del Sacro Cuore, 00168, Rome, Italy; 2 Fondazione Policlinico Universitario «A. Gemelli» IRCCS, 00168, Rome, Italy; 3 Department of Medicine and Surgery, LUM University, 70100, Casamassima, Italy

**Keywords:** Nutrition, hypertension, diabetes, cardiovascular disease, elderly

## Abstract

**Objectives:**

To examine the relationships between protein intake from various food sources and cardiometabolic risk markers in Italian older adults.

**Design:**

Cross-sectional study. Setting: Unconventional settings across Italy (e.g., exhibitions, health promotion campaigns).

**Participants:**

People 65+ years who provided a written informed consent.

**Measurements:**

Blood pressure (BP), blood glucose, total blood cholesterol, and anthropometric indices were assessed. Daily protein intake was estimated for 12 food items listed in a food frequency questionnaire.

**Results:**

Three-thousand four-hundred twenty-four older adults (mean age: 72.7 ± 5.7 years; 55% women) were included in the study. Results of linear regression analysis indicated that protein intake from several food sources was negatively associated with BP, waist and hip circumferences, and waist-to-hip ratio in both sexes. Blood glucose levels were inversely associated with many protein sources in women. Positive associations were observed between some protein sources and total blood cholesterol in both men and women.

**Conclusion:**

Our findings suggest that dietary protein is differentially associated with cardiometabolic risk factors depending on sex and food sources.

## Introduction

**C**ardiovascular disease (CVD) is the leading cause of death worldwide (1). The prevalence of CVD increases with age, affecting more than 70% of those 60 years and older (2). High blood pressure (BP), abnormal glucose metabolism, hyperlipidemia (3), and excess adiposity are common in advanced age, and contribute to cardiometabolic dysregulation (4).

Nutritional habits have a key role in the prevention and treatment of cardiometabolic diseases (5). Protein intake is a major determinant of muscle health by providing amino acids to sustain muscle anabolism (6). The amount of amino acids required to properly stimulate muscle protein synthesis is still under discussion. Experts in the field have suggested that older adults require a higher protein intake than young individuals to overcome an age-related state of anabolic resistance (7). Whether a high-protein diet may negatively affect cardiometabolic health in old age is presently unclear.

The impact of protein intake on cardiometabolic parameters is under intense debate, with studies reporting positive, negative, or null relationships (8, 9, 10, 11). However, it should be noted that most studies were conducted in small samples of adults from different age groups, with only few investigations that specifically examined older adults. Moreover, dietary protein is consumed together with other macro- and micronutrients that may themselves affect CVD risk. The assessment of the impact of protein food sources on cardiometabolic risk factors may allow a better appraisal of the relationship between dietary protein and cardiometabolic health. This information, in turn, is instrumental in guiding the prescription of dietary plans that enhance muscle health while protecting the cardiovascular system in old age. The present study was, therefore, conducted to test the association between protein from a variety of food sources and BP, blood glucose, and total blood cholesterol in a large and relatively unselected sample of Italian older adults.

## Methods

Data of the present retrospective study were extracted from the database of the Longevity Check-up 7+ (Lookup 7+) project. Participant recruitment was carried out during public events (e.g., exhibitions, health promotion campaigns) to allow enrolment of individuals resembling the general population. The manuscript was prepared in compliance with the STrengthening the Reporting of OBservational studies in Epidemiology (STROBE) guidelines for observational studies (12). The Lookup 7+ protocol was approved by the Ethics Committee of the Università Cattolica del Sacro Cuore (protocol #: A.1220/CE/2011) and each participant provided written informed consent prior to enrolment.

### Participants and data collection

Between 1 June 2015 and 31 October 2021, 13,515 community-dwelling individuals 18 years and older were recruited. Exclusion criteria were inability or unwillingness to provide written informed consent, self-reported pregnancy, and inability to complete the physical function tests in accordance with the study protocol. Only those who were 65 years or older and had a body mass index (BMI) of at least 18.5 kg/m^2^ were included in the present study (n = 3424).

Following a structured interview to collect information on lifestyle habits, anthropometric parameters and BP were measured. An anthropometric tape was used to measure waist and hip circumferences. Participants were asked to remain standing with their feet together, head straight, eyes forward, and arms relaxed along the trunk. The waist circumference was measured at the mid-point between the last floating rib and the highest point of the iliac crest. The hip circumference was taken at the highest point of the buttocks. The BMI was calculated as the ratio between body weight (kg) and the square of height (m^2^). BP measurements were obtained after resting for 5 min in a seated position, with 30s intervals between cuff inflations using an oscillometric monitor (Omron M6 electronic sphygmomanometer, Omron, Kyoto, Japan) in a dedicated room. The cuff was selected according to arm size of the test person. An average of three measurements was used for the analysis. A portable reflectometric system (MultiCareIn, Biomedical Systems International Srl, Florence, Italy) was used to measure blood glucose and total blood cholesterol concentrations (md/dL) from capillary blood samples. Participants were asked if they were fasted for at least 8 h. A food frequency questionnaire (FFQ) adapted from Morin et al. (13) was used to collect information on how many times a standardized portion size of a list of 12 foods was consumed during the previous week. Food items included meat, meat derivatives, fish, eggs, milk, cheese, yogurt, pasta, bread, rice, vegetables, and cereals. Portion size was estimated based on the Italian standard portion reference (13). The daily intake of protein from the various food sources was determined by multiplying the frequency of consumption of a particular food item by the protein amount of its standard portion, then dividing by seven (the days of a week). The mean protein intake was estimated by summing all applicable items. Smoking status was divided into two categories: current smokers (i.e., those who have smoked 100 or more cigarettes in their lifetime) and non-current smokers. Walking for 30 or more minutes, at least twice a week over the previous year was operationalized as regular walking (14). Information on the use of antihypertensive, cholesterol-lowering, and antidiabetic drugs was collected through self-report.

### Statistical Analysis

The Shapiro-Wilk test was used to determine whether the distribution of data was normal. Continuous variables are summarized using mean ± standard deviation (SD), while categorical variables are reported as absolute numbers and percentages. Independent t-tests and χ-square statistics were used to examine differences in continuous and categorical variables, respectively, according to sex. Unadjusted β, linear regression, was calculated for the relationship between protein consumption from specific food sources and cardiometabolic risk factors. Linear regression was corrected for model 1 and 2. Model 1 included age, sex, BMI, energy intake corresponding to the food source analyzed as a dependent variable, walking activity, active smoking, fasting state (blood glucose), and antihypertensive (for systolic [SBP] and diastolic BP [DBP]), cholesterol-lowering (for total blood cholesterol), and antidiabetic (for blood glucose) drugs. Model 1 also included sodium, potassium, calcium, and magnesium by protein sources for SBP and DBP. Model 2 included variables of model 1 plus total energy, carbohydrate, and fat intakes. Variables to adjust the model were selected based on their association with cardiometabolic risk. Significance was set at 5% (p < 0.05) for all tests. All p-values were determined by two-tailed tests. All analyses were performed using the SPSS software (version 23.0, SPSS Inc., Chicago, IL, USA).

## Results

### Participants Characteristics

Data from 3424 older adults were analyzed for the present study. The main characteristics of study participants by sex are shown in Table 1. Women (n = 1883, 55%) had a mean age of 72.4 ± 5.5 years and an average BMI of 26.1 ± 4.4 kg/m^2^. The mean SBP and DBP values were slightly below the cutoff levels for hypertension (15), whereas total blood cholesterol and waist circumference were borderline (1). Mean waist-to-hip ratio indicated abdominal obesity (1). Women ingested protein primarily from cheese, meat, and fish (144 g/day, ≥ 0.6 g/kg/day), representing 52.3% of the total body weight-adjusted protein intake. Secondary sources, which accounted for 42.0% of overall protein intake, included pasta, bread, yogurt, vegetables, meat derivatives, and milk, provided 115 g of protein/day (0.2 g/kg/day). Protein from eggs, cereals, and rice represented 5.7% of total protein intake (15.5 g/day, ≤0.1 g/kg/day). Cheese, bread, and pasta accounted for more than half (53.8%) of total energy intake.

**Table 1:** Main Characteristics of Study Participants According to Sex (n = 3424)

	**Women (n = 1883)**	**Men (n = 1541)**
Age, years	72.4 ± 5.5	73.0 ± 5.8
Body mass index, kg/m^2^	26.1 ± 4.4	26.8 ± 3.5
Systolic blood pressure, mmHg	129.4 ± 16.5	132.1 ± 15.4
Diastolic blood pressure, mmHg	75.3 ± 9.6	76.6 ± 9.4
Blood glucose, mg/dL	105.2 ± 22.4	108.8 ± 28.5*
Total blood cholesterol, mg/dL	204.2 ± 32.7	194.6 ± 32.6
Waist circumference, cm	89.4 ± 12.7	99.0 ± 10.3*
Hip circumference, cm	103.1 ± 10.2	102.8 ± 7.8*
Waist-to-hip ratio	0.86 ± 0.07	0.96 ± 0.08
Current smokers, yes	234, 12.4	220, 14.2
Walking, yes	901, 47.8	814, 52.8*
Antihypertensive drug (s), yes	957, 50.8	876, 56.8*
Cholesterol-lowering drug(s), yes	673, 25.7	457, 30.0*
Antidiabetic drug(s), yes	104, 5.5	180, 11.6*
Protein intake by food sources, g/day		
Meat	43.0 ± 26.1	45.7 ± 26.6
Meat derivatives	17.6 ± 18.7	19.4 ± 19.5
Fish	46.2 ± 31.1	44.9 ± 27.3*
Eggs	8.0 ± 5.7	7.9 ± 6.2
Milk	15.9 ± 13.1	15.8 ± 13.2
Cheese	54.9 ± 39.6	56.5 ± 39.0
Yogurt	19.6 ± 20.9	15.2 ± 20.5*
Pasta	23.1 ± 12.9	27.8 ± 11.6*
Bread	20.7 ± 10.6	22.7 ± 9.3*
Rice	2.3 ± 1.8	2.3 ± 1.8
Vegetables	18.1 ± 14.2	19.5 ± 13.7*
Cereals	5.2 ± 9.0	4.0 ± 8.0*
Protein intake by food sources, g/kg of body weight/day		
Meat	0.67 ± 0.42	0.59 ± 0.36*
Meat derivatives	0.27 ± 0.29	0.24 ± 0.25*
Fish	0.73 ± 0.53	0.58 ± 0.37*
Eggs	0.12 ± 0.09	0.10 ± 0.08*
Milk	0.25 ± 0.21	0.20 ± 0.17*
Cheese	0.86 ± 0.64	0.73 ± 0.51*
Yogurt	0.31 ± 0.34	0.19 ± 0.26*
Pasta	0.36 ± 0.22	0.36 ± 0.16
Bread	0.32 ± 0.18	0.29 ± 0.13*
Rice	0.03 ± 0.03	0.03 ± 0.03
Vegetables	0.29 ± 0.24	0.25 ± 0.18*
Cereals	0.08 ± 0.14	0.05 ± 0.10*


Data are expressed as mean ± standard deviation or absolute numbers, percentages.

*P<0.05 refers to significant differences between men and women.

Men had a mean age of 73.1 ± 5.8 years and an average BMI 26.8 ± 3.5 kg/m2. The mean SPB value was higher than

the cutoff for hypertension. Average waist circumference and waist-to-hip ratio indicated that men participants were at high cardiovascular risk. DBP values and total blood cholesterol levels were within their normal range. Major protein sources (147.1 g/day, ≥ 0.6 g/kg/ day) were cheese, meat, and fish, which altogether represented 52.3% of total intake. Pasta, bread, vegetables, meat derivatives, milk, and yogurt contributed to 45.5% of total protein consumption (120.4 g/day). Smaller amounts of protein were ingested from eggs, cereals, and rice (2.2%). More than 50% of daily energy intake was obtained from cheese, which accounted for 52.8% of the total. Milk, vegetables, pasta, and fish contributed to 41.7% of total energy intake.

Comparisons between sexes revealed that men had significant higher glucose levels, larger waist circumference, smaller hip circumference, were more frequently physically active, and were more frequently on antihypertensive, cholesterol-lowering, and antidiabetic drugs. Women consumed more protein from fish, yogurt, and cereals, whereas men had a higher ingestion of protein from pasta, bread, and vegetables. When adjusted for body weight, women consumed significantly more protein from meat, meat derivates, fish, eggs, milk, cheese, yogurt, bread, vegetables, and cereals. No other significant differences were observed between sexes.

### Association Between Protein Intake by Food Sources and Cardiometabolic Markers in Women

Results of the linear regression for the association between protein intake by food sources and BP, blood glucose, total blood cholesterol, and anthropometric parameters in women are shown in Tables 2 and 3. Protein intake from pasta, bread, and vegetables was negatively associated with SBP. DBP was also negatively associated with protein consumption from meat, meat derivates, fish, milk, cheese, and yogurt. A higher ingestion of protein from meat, meat derivates, fish, pasta, and bread was inversely associated with blood glucose levels. In contrast, a positive association was noted between total blood cholesterol and protein from meat, meat derivates, fish, milk, pasta, rice, and vegetables. All protein sources were negatively associated with waist and hip circumferences, whereas waist-to-hip ratio was only significantly associated with protein intake from meat, meat derivates, fish, eggs, yogurt, past, bread, rice, and cereals.

**Table 2:** Association Between Protein Intake by Food Sources and Blood Pressure, Blood Glucose and Total Blood Cholesterol in Older Women

	**Unadjusted β (95% CI)**	**Adjusted§ β (95% CI)**	**Adjusted† β (95% CI)**	**Unadjusted β (95% CI)**	**Adjusted§ β (95% CI)**	**Adjusted† β (95% CI)**	**Unadjusted β (95% CI)**	**Adjusted§ β (95% CI)**	**Adjusted† β (95% CI)**	**Unadjusted β (95% CI)**	**Adjusted§ β (95% CI)**	**Adjusted† β (95% CI)**
	**Systolic blood pressure, mmHg**			**Diastolic blood pressure, mmHg**			**Blood glucose, mg/dL**			**Total blood cholesterol, mg/dL**		
Meat	0.4 (−1.29, 2.27)	−4.6 (−10.1, 0.8)	−4.7 (−10.2, 0.7)	−1.1 (−2.5, 0.1)	−7.7 (−12.6, −3.0)*	−5.7 (−8.9, −2.4)*	−1.4 (−3.8, 0.8)	−0.7 (−3.0, 1.5)	−11.2 (−17.9, −4.4)*	−0.4 (−4.0, 3.0)	0.2 (−3.2, 2.7)	17.7 (7.0, 28.4)*
Meat derivatives	1.4 (−1.1, 3.9)	−7.1 (−18.4, 4.1)	−6.4 (−17.6, 4.7)	−0.2 (−1.7, 1.2)	−10.3 (−16.9,-3.7)*	−10.2 (−16.8,-3.6)*	−3.0 (−6.4, 0.3)	−1.9 (−5.2, 1.3)	−14.1 (−27.6, −0.5)*	0.4 (−4.6, 5.4)	0.8 (−4.1, 5.8)	21.8 (0.6, 43.1)*
Fish	0.18 (−1.2, 1.6)	−3.8 (−8.9, 1.2)	−3.6 (−8.7, 1.4)	0.02 (−0.8, 0.8)	−4.9 (−7.9, -1.9)*	−4.9 (−7.9, −1.9)*	−2.5 (−4.4, −0.6)*	−2.2 (−4.0, −0.3)*	−7.7 (−13.7, −1.7)*	0.9 (−1.8,3.8)	0.7 (−2.1, 3.5)	14.4 (4.8, 23.9)*
Eggs	2.0 (−6.1, 10.1)	4.1 (−34.6, 42.9)	1.1 (−37.7, 40.1)	−2.6 (−7.4, 2.1)	−2.0 (−25.0, 20.8)	−3.9 (−26.9, 19.1)	−1.07 (−11.7, 9.5)	−1.4 (−12.0, 9.2)	−47.7 (−96.0, 0.4)	5.5 (−10.3, 21.5)	−0.1 (−16.1, 15.9)	16.0 (−59.2, 91.4)
Milk	−0.31 (−3.8, 3.2)	−6.7 (−20.5, 7.0)	−6.8 (−20.6, 6.9)	−1.3 (−3.4, 0.7)	−11.4 (−19.6, −3.3)*	−11.8 (−20.0, −3.6)*	−0.5 (−5.2,4.1)	−1.0 (−5.6, 3.4)	−16.0 (−32.9, 0.9)	1.6 (−5.3, 8.6)	4.3 (−2.5, 11.3)	30.4 (3.8, 57.0)*
Cheese	−0.40 (−1.5, 0.7)	−3.7 (−9.6, 2.4)	−3.6 (−9.5, 2.1)	−0.8 (−1.5, −0.8)	−3.6 (−7.0, −0.1) *	−3.4 (−6.9, −0.4) *	−0.6 (−2.2, 0.9)	−0.1 (−1.7, 1.3)	−4.3 (−11.5, 2.8)	3.0 (0.6, 5.3)*	2.8 (0.0, 4.7)*	6.2 (−5.0, 17.5)
Yogurt	0.04 (−2.1, 2.2)	−6.0 (−15.6, 3.5)	−5.6 (−15.2, 3.9)	−0.6 (−1.9, 0.6)	−7.6 (−13.3, −1.9)*	−7.6 (−13.3, −1.9)*	−2.6 (−5.6, 0.3)	−1.3 (−4.9, 1.5)	−5.5 (−17.3, 6.1)	1.6 (−2.8, 6.0)	1.0 (−3.3, 5.4)	14.6 (−4.0, 33.4)
Pasta	−0.4 (−3.8, 3.0)	−10.9 (−21.3, −0.5)*	−10.6 (−21.0, −0.2)*	−1.5 (−3.5, 0.4)	−11.2 (−17.4, −5.1)*	−11.5 (−17.7, −5.4)*	4.0 (−0.5, 8.5)	3.0 (−1.3, 7.5)	−13.3 (−26.1, −0.4)*	1.4 (−5.3, 8.2)	2.8 (−3.8, 9,5)	40.3 (20.2, 60.4)*
Bread	−3.8 (−8.0, 0.4)	−15.6 (−27.7, −3.5)*	−15.4 (−27.5, −3.2)*	−2.4 (−4.9, 0.5)	−15.3 (−22.5, −8.2)*	−15.5 (−22.7, −8.4)*	−0.4 (−5.9, 5.1)	1.0 (−4.3, 6.4)	−20.7 (−35.6, −5.9)*	6.8 (−1.5, 15.2)	6.6 (−1.6, 14.9)	28.7 (5.2, 52.1)*
Rice	3.6 (−21.3, 28.7)	−54.7 (−152.0, 42.5)	−51.9 (−149.6, 45.8)	−1.0 (−15.7, 13.6)	3.2 (1.2, 5.1)	−94.9 (−152.8, −37.0)*	−33.0 (−66.0, −0.1)*	−26.3 (−58.3, 5.7)	−60.2 (−180.5, 60.1)	51.3 (1.6, 101.0)*	42.0 (−7.1, 91.3)	204.8 (14.3, 395.2)*
Vegetables	0.1 (−3.0, 3.3)	−15.0 (−26.7, −3.4)*	−14.2 (−26.0, −2.5)*	0.2 (−1.6, 2.1)	−10.3 (−17.2, −3.3)*	−10.4 (−17.4, −3.5)*	−3.7 (−7.8, 0.4)	−3.3 (−7.4, 0.7)	−6.9 (−21.4, 7.5)	5.3 (−1.0, 11.6)	4.6 (−1.5, 10.9)	31.2 (8.0, 54.3)*
Cereals	−4.5 (−9.7, 0.5)	−21.0 (−49.3, 7.2)	−19.5 (−47.9, 8.7)	−3.5 (−6.5, −0.5)	−16.4 (−33.2, 0.2)	−16.3 (−33.1, 0.4)	−5.2 (−12.1, 1.6)	−2.2 (−8.8, 4.4)	−24.9 (−60.2, 10.2)	7.0 (−3.1, 17.1)	3.9 (−6.0, 13.8)	15.5 (−71.1, 40.0)


§ Model 1= age, body mass index, energy intake corresponding to the food source used as a dependent variable, micronutrients (i.e., sodium, potassium, calcium, and magnesium) by food sources, walking, smoking habit, fasting state (blood glucose), and antihypertensive (for systolic (SBP) and diastolic BP (DBP)), cholesterol-lowering (for total blood cholesterol), and antidiabetic (for blood glucose) drugs. † Model 2= Model 1+ total energy intake, carbohydrate and fat ingestion. * p < 0.05. Abbreviation: CI, confidence interval.

**Table 3:** Association Between Protein Intake and Anthropometric Parameters in Older Women

	**Unadjusted β (95% CI)**	**Adjusted§ β (95% CI)**	**Adjusted† β (95% CI)**	**Unadjusted β (95% CI)**	**Adjusted§ β (95% CI)**	**Adjusted† β (95% CI)**	**Unadjusted β (95% CI)**	**Adjusted§ β (95% CI)**	**Adjusted† β (95% CI)**
	**Waist circumference, cm**			**Hip circumference, cm**				**Waist-to-hip ratio**	
Meat	−3.5 (−5.5, −1.5)*	−0.7 (−1.9, 0.4)	17.5 (−22.5, -12.5)*	−2.5 (4.1, -0.9)*	−0.06 (−0.96, 0.82)	−11.6 (−15.4, -7.9)*	−0.01 (−0.03, -0.00)*	−0.01 (−0.02, 0.00)	−0.08 (−0.12, -0.03)*
Meat derivatives	−1.1 (−4.1, 1.8)	−1.4 (−4.3, 1.5)	−93.6 (−103.9, -83.3)*	−4.0 (−6.4, -1.7)*	4.2 (−6.6, -1.9)*	-78.4 (−86.6, -70.1)*	0.02 (0.01, 0.04)*	0.03 (0.01, 0.04)*	-0.25 (−0.32, -0.18)*
Fish	−5.5 (−7.1, −4.0)*	−0.5 (−1.5, 0.4)	−15.2 (−19.3, -11.0)*	−4.1 (−5.3, -2.9)*	0.1 (−0.6, 0.8)	-12.7 (−15.7, -9.6)*	-0.02 (−0.03, -0.01)*	-0.01 (−0.02, 0.00)	-0.05 (−0.09, -0.01)*
Eggs	−25.8 (−33.8, −17.7)*	−7.4 (−12.5, −2.2)*	−93.1 (−117.3, −69.0)*	−19.5 (−26.0, −13.0)*	−3.3 (−7.1, 0.5)	−68.3 (−86.2, −50.4)*	−0.09 (−0.14, −0.04)*	−0.05 (−0.10, −0.00)*	−0.38 (−0.60, −0.16)*
Milk	−3.4 (−7.3, 0.4)	−1.2 (−3.6, 1.1)	−34.9 (−46.8, −23.0)*	−3.2 (−6.4, −0.1)*	−1.2 (−2.9, 0.5)	−29.9 (−38.7, −21.1)*	−0.01 (−0.03, 0.02)	−0.00 (−0.02, 0.02)	−0.09 (−0.20, 0.01)
Cheese	−2.6 (−3.9, −1.3)*	−0.9 (−1.7, 0.1)	−9.7 (−13.4, −5.9)*	−2.0 (−3.0, −0.9)*	−0.4 (−1.0, 0.1)	−8.2 (−11.0, −5.4)*	−0.01 (−0.02, −0.00)*	−0.01 (−0.02, 0.00)	−0.03 (−0.66, 0.01)
Yogurt	−6.8 (−9.2, −4.4)*	−6.5 (−8.9, −4.2)*	−77.6 (−86.1, −69.1)*	−5.0 (−6.9, −3.0)*	4.8(−6.7, −2.8)*	−64.5 (−71.3, −57.7)*	−0.03 (−0.04, −0.01)*	−0.03 (−0.04, −0.01)*	−0.22 (−0.28, −0.17)*
Pasta	−12.8 (−16.5, −9.2)*	−1.6 (−3.9, 0.7)	−37.9 (−47.1, −28.6)*	−12.8 (−15.7, −9.9)*	−3.4 (−5.1, −1.6)*	−27.9 (−34.6, −20.9)*	−0.02 (−0.04, 0.00)	0.02 (−0.01, 0.04)	−0.15 (−0.24, −0.07)*
Bread	−15.7 (−20.3, −11,2)*	−3.2 (−6.1, −0.3)*	−46.4 (−57.9, −34.9)*	−14.0 (−17.6, −10.4)*	−3.5 (−5.7, −1.5)*	−37.3 (−45.8, −28.8)*	−0.04 (−0.06, −0.01)*	0.00 (−0.03, 0.03)	−0.15 (−0.26, −0.04)*
Rice	−83.3 (−109.9, −56.7)*	−15.8 (−32.5, 0.9)	−216.0 (−301.2, −130.8)*	−60.1 (−81.6, −38.5)*	−1.8 (−14.3, 10.6)	−161.5 (−224.6, −98.4)*	−0.32 (−0.47, −0.16)*	−0.15 (−0.30, 0.00)	−0.94 (−1.70, −0.10)*
Vegetables	−12.7 (−15.9, −9.4)*	−2.9 (−5.0, −0.9)*	−21.7 (−31.0, −12.4)*	−10.8 (−13.4, −8.2)*	−2.5 (−4.1, −1.0)*	−18.6 (−25.5, −11.8)*	−0.03 (−0.05, −0.01)*	−0.01 (−0.03, 0.01)	−0.07 (−0.15, 0.01)
Cereals	−14.2 (−20.4, −8.1)*	−12.8 (−18.8, − 6.7)*	−155.9 (−186.3, −125.5)*	−10.1 (−15.0, −5.2)*	−9.1 (−14.0, −4.2)*	−136.2 (−160.6, −111.8)*	−0.05 (−0.09, −0.02)*	−0.05 (−0.09, −0.01)*	−0.402 (−0.587, −0.216)*


§ Model 1= age, body mass index, energy intake corresponding to the food source used as a dependent variable, walking, and smoking habit. † Model 2= Model 1+ total energy intake, carbohydrate and fat ingestion. * p < 0.05. Abbreviation: CI, confidence interval.

### Association Between Protein Intake by Food Sources and Cardiometabolic Markers in Men

Results of the linear regression for the association between protein intake by food sources and BP, blood glucose, total blood cholesterol, and anthropometric parameters in men are shown in Tables 4 and 5. SBP and DBP were negatively associated with protein consumption from meat, fish, eggs, cheese, bread, rice, and vegetables. Protein intake from meat derivatives, milk, and pasta was negatively associated with DBP. Total blood cholesterol levels were positively associated with protein ingestion from meat derivates, fish, milk, yogurt, pasta, bread, and cereals. All protein sources were negatively associated with waist and hip circumferences, as well as waist-to-hip ratio.

**Table 4:** Association Between Protein Intake by Food Sources and Blood Pressure, Blood Glucose and Total Blood Cholesterol in Older men

	**Unadjusted β (95% CI)**	**Adjusted§ β (95% CI)**	**Adjusted† β (95% CI)**	**Unadjusted β (95% CI)**	**Adjusted§ β (95% CI)**	**Adjusted† β (95% CI)**	**Unadjusted β (95% CI)**	**Adjusted§ β (95% CI)**	**Adjusted† β (95% CI)**	**Unadjusted β (95% CI)**	**Adjusted§ β (95% CI)**	**Adjusted† β (95% CI)**
	**Systolic blood pressure, mmHg**			**Diastolic blood pressure, mmHg**			**Blood glucose, mg/dL**			**Total blood cholesterol, mg/dL**		
Meat	0.65 (−1.5, 2.8)	−8.4 (−16.1, −0.83)*	−8.5 (−16.1, −0.8)*	−1.1 (−2.5, 0.1)	−7.7 (−12.6, −3.0)*	−7.7 (−12.3, −3.0)*	4.0 (0.0, 8.0)	3.0 (−0.7, 6.8)	2.6 (−11.2, 16.5)	−3.5 (−8.1, 1.0)	−3.7 (−8.3, 0.8)	10.5 (−5.3, 26.3)
Meat derivatives	2.76 (−0.3, 5.8)	−11.9 (−26.8, 3.0)	−12.4 (−27.4, 2.5)	0.4 (−1.5, 2.3)	−14.8 (−24.0, −5.7)*	−15.0 (−24.1, −5.8)*	7.8 (2.1, 13.5)	4.4 (−0.9, 9.9)	1.4 (−26.1, 29.0)	4.2 (−2.5, 11.0)	5.2 (−1.4, 12.0)	37.3 (5.0, 69.6)*
Fish	−0.93 (−3.0, 1.1)	−10.0 (−18.0, −2.0)*	−9.6 (−17.6, −1.6)*	−1.1 (−2.4, 0.1)	−7.0 (−11.9, −2.1)*	−7.0 (−11.9, −2.2)*	−2.0 (−5.9, 1.8)	−3.3 (−6.9, 0.3)	−7.4 (−22.1, 7.2)	1.5 (−2.9, 6.1)	1.3 (−3.1, 5.8)	20.8 (3.9, 37.6)*
Eggs	−3.3 (−12.9, 6.2)	−88.5 (−130.0, −46.4)*	−88.5 (−131.0, −46.6)*	−2.3 (−8.1, 3.5)	−58.1 (−83.8, −32.5)*	−28.2 (−84.0, −32.5)*	2.6 (−14.5, 19.8)	3.0 (−13.3, 19.4)	7.9 (−66.6, 82.6)	−1.7 (−22.0, 18.4)	−0.4 (−20.7, 19.8)	37.5 (−49.5, 124.7)
Milk	1.3 (−3.0, 5.7)	−15.9 (−35.8, 3.8)	−15.5 (−35.4, 4.3)	−0.4 (−3.1, 2,1)	−17.7 (−29.8, −5.6)*	−17.4 (−29.5, −5.3)*	5.4 (−2.5, 13.5)	−1.5 (−9.2, 6.2)	−21.5 (−57.7, 14.6)	1.2 (−8.2, 10.6)	4.9 (−4.4, 14.3)	61.0 (19.0, 103.0)*
Cheese	−0.02 (−1.5, 1.5)	−7.6 (−13.7, −1.5)*	−7.8 (−13.9, −1.7)*	−0.9 (−1.1, 0,7)	−7.3 (−11.0, −3.6)*	−7.3 (−11.0, −3.6)*	0.9 (−1.8, 3.7)	0.1 (−2.5, 2.7)	1.8 (−9.2, 13.0)	0.75 (−2.5, 4.0)	1.2 (−1.9, 4.5)	10.7 (−2.0, 23.6)
Yogurt	3.0 (0.1, 5.9)*	−10.9 (−28.0, 6.0)	−10.7 (−27.8, 6.3)	0.5 (−1.2, 2.3)	−9.2 (−19.6, 1.1)	−9.2 (−19.6, 1.1)	−2.4 (−7.7, 2.9)	−4.2 (−9.2, 0.8)	30.2 (−0.8, 61.2)	3.6 (−2.5, 9.8)	3.1 (−3.0, 9.3)	36.2 (0.7, 71.8)*
Pasta	−1.4 (−6.2, 3,2)	−12.6 (−26.5, 1.2)	−12.1 (−26.1, 1.7)	−4.1 (−7.0, 1.2)	−15.0 (−23.4, −6.5)*	−14.9 (−23.4, −6.4)*	5.5 (−3.0, 14.1)	2.9 (−5.3, 11.1)	−9.3 (−34.7, 15.9)	9.7 (−0.3, 19.8)	12.2 (2.1, 22.3)*	47.2 (18.1, 76.2)*
Bread	0.58 (−5.4, 6.5)	−22.0 (−39.1, −4.8)*	−21.3 (−38.5, −4.1)*	−0.3 (−3.9, 3,3)	−22.7 (−33.1, −12.3)*	−22.5 (−32.9, −12.0)*	−4.4 (−15.4, 6.5)	−4.5 (−14.9, 5.9)	−13.4 (−45.0, 18.1)	11.4 (−1.4, 24.4)	13.7 (0.9, 25.6)*	43.1 (6.6, 79.5)*
Rice	−4.9 (−36.3, 26.4)	−253.9 (−398.4, −109.5)*	0.72 (0.29, 1.16)*	4.8 (−14.3, 24.0)	−173.8 (−261.7, −85.9)*	−172.8 (−261.2, −84.4)*	−47.8 (−104.6, 8.9)	−45.6 (−100.2, 8.8)	−49.2 (−314.5, 216.1)	−11.8 (−78.7, 55.0)	8.1 (−58.7, 75.0)	122.1 (−181.0, 425.3)
Vegetables	−0.76 (−4.9, 3.4)	−20.8 (−38.4, −3.2)*	−20.0 (−37.6, −2.3)*	−3.0 (−5.6, −0.4)*	−16.8 (−27.5, −6.1)*	−16.6 (−27.4, −5.9)*	4.1 (−3.5, 11.8)	1.1 (−6.1, 8.5)	−9.7 (42.1, 22.5)	−2.4 (−11.4, 6.5)	−0.3 (−9.3, 8.7)	32.7 (−4.8, 70.2)
Cereals	−2.8 (−10.3, 4.6)	−25.1 (−69.8, −19.5)*	−25.6 (−70.3, 19.2)	−0.3 (−4.9, 4,2)	−18.3 (−45.5, 8.9)	−18.6 (−45.8, 8.6)*	−10.6 (−24.2, 2.9)	−11.2 (−24.1, 1.5)	27.0 (−54.0, 108.1)	18.1 (2.2, 34.1)*	16.4 (0.7, 32.4)*	137.1 (42.9, 231.2)*


§ Model 1= age, body mass index, energy intake corresponding to the food source used as a dependent variable, micronutrients (i.e., sodium, potassium, calcium, and magnesium) by food sources, walking, smoking habit, fasting state (blood glucose), and antihypertensive (for systolic (SBP) and diastolic BP (DBP)), cholesterol-lowering (for total blood cholesterol), and antidiabetic (for blood glucose) drugs. † Model 2= Model 1+ total energy intake, carbohydrate and fat ingestion; * p < 0.05. Abbreviation: CI, confidence interval.

**Table 5:** Association Between Protein Intake and Anthropometric Parameters in Older men.

	**Unadjusted β (95% CI)**	**Adjusted§ β (95% CI)**	**Adjusted† β (95% CI)**	**Unadjusted β (95% CI)**	**Adjusted§ β (95% CI)**	**Adjusted† β (95% CI)**	**Unadjusted β (95% CI)**	**Adjusted§ β (95% CI)**	**Adjusted† β (95% CI)**
	**Waist circumference, cm**				**Word**			**Waist-to-hip ratio**	
Meat	−2.3 (−4.3, −0.3)*	−2.6 (−4.5, −0.6)*	−65.7 (−71.3, −60.1)*	−2.0 (−3.5, −0.3)*	−2.1 (−3.5, −0.6)*	−46.8 (−-51.2, −42.4)*			−0.20 (−0.26, −0.15)*
Meat derivatives	2.9 (−0.01, 5.8)	3.4 (0.4, 6.3)*	−113.5 (−127.0, −99.9)*	0.8 (−1.3, 3.0)	0.6 (−1.5, 2.8)	−78.8 (−89.4, −68.2)*	−0.01 (−0.02, 0.11)	−0.01 (−0.02, 0.01)	−0.37 (−0.49, −0.24)*
Fish	−5.7 (−7.7, −3.7)*	−5.2 (−7.2, −3.2)*	−71.4 (−77.5, −65.3)*	−5.4 (−6.9, −3.9)*	−5.2 (−6.7, −3.7)*	−46.3 (−51.4, −41.2)*	0.02 (0.00, 0.05)*	0.03 (0.00, 0.05)*	−0.28 (−0.34, −0.22)*
Eggs	−18.6 (−26.8, −10.4)*	−18.5 (−26.7, −10.2)*	−279.1 (−309.4, −248.8)*	−13.1 (−19.3, −6.8)*	−13.4 (−19.7, −7.2)*	−192.6 (−216.7, −168.6)*	0.00 (−0.016, 0.02)	0.00 (−0.01, 0.02)	−0.94 (−1.23, −0.66)*
Milk	−7.5 (−11.5, −3.5)*	−7.6 (−11.6, −3.6)*	−149.2 (−164.9, −133.5)*	−3.6 (−6.7, −0.6)*	−3.8 (−6.9, −0.8)*	−105.0 (−117.5, −92.6)*	−0.07 (−0.13, 000)	−0.07 (−0.13, −0.00)*	−0.50 (−0.64, −0.35)*
Cheese	−0.6 (−2.0, 0.7)	−0.3 (−1.7, 1.0)	−45.2 (−49.6, −40.8)*	−0.4 (−1.5, 0.6)	−0.5 (−1.5, 0.5)	−36.9 (−36.9, −30.1)*	−0.04 (−0.07, −0.00)*	−0.04 (−0.07, −0.01)*	−0.13 (−0.17, −0.08)*
Yogurt	−3.0 (−5.7, −0.3)*	−2.0 (−4.7, 0.6)	−82.2 (−95.9, −68.4)*	−1.0 (−3.0, 1.0)	−0.7 (−2.7, 1.3)	−59.0 (−69.6, −48.3)*	−0.00 (−0.01, 0.01)	−0.01 (−0.02, 0.01)	−0.29 (−0.41, −0.17)*
Pasta	−16.6 (−20.8, −12.4)*	−16.0 (−20.3, −11.8)*	−122.3 (−131.7, −112.9)*	−14.4 (−17.5, −11.2)*	−14.4 (−17.6, −11.2)*	−84.9 (−92.6, −77.2)*	−0.02 (−0.04, 0.00)	−0.02 (−0.04, 0.00)	−0.41 (−0.51, −0.32)*
Bread	−18.9 (−24.2, −13.6)*	−19.6 (−24.9, −14.3)*	−154.6 (−167.2, −142.0)*	−12.9 (−16.9, −8.8)*	−13.3 (−17.3, −9.2)*	−111.2 (−121.2, −101.1)*	−0.03 (−0.06, 0.01)	−0.03 (−0.07, 0.01)	−0.48 (−0.61, −0.36)*
Rice	−71.8 (−98.6, −45.0)*	−72.7 (−99.7, −45.6)*	−1093.5 (−1203.3, −983.8)*	−49.7 (−70.1, −29.2)*	−51.4 (−72.1, −30.7)*	−766.0 (−853.7, −678.3)*	−0.06 (−0.10, −0.02)*	−0.08 (−0.13, −0.04)*	−3.7 (−4.7, −2.6)*
Vegetables	−11.4 (−15.1, −7.7)*	−11.4 (−15.1, −7.7)*	−142.0 (−155.5, −128.4)*	−7.1 (−9.9, −4.3)*	−7.0 (−9.9, −4.2)*	−98.6 (−109.5, −87.7)*	−0.26 (−0.48, −0.05)*	−0.29 (−0.51, −0.07)*	−0.48 (−0.61, −0.35)*
Cereals	−11.6 (−19.5, −3.7)*	−11.3 (−19.0, −3.5)*	−213.6 (−259.0, −168.1)*	−4.3 (−10.3, 1.6)	−3.6 (−9.5, 2.2)	−165.6 (−-200.2, −130.9)*	−0.05 (−0.08, −0.02)*	−0.06 (−0.09, −0.03)*	−0.54 (−0.92, −0.16)*


§ Model 1= age, body mass index, energy intake corresponding to the food source used as a dependent variable, walking, and smoking habit. † Model 2= Model 1+ total energy intake, carbohydrate and fat ingestion.

## Discussion

The main findings of the present study indicate that dietary protein is differentially associated with cardiometabolic risk factors depending on sex and food sources. Several protein sources were negatively associated with BP, waist and hip circumferences, and waist-to-hip ratio in both sexes. Significant negative associations were also observed for several protein sources and blood glucose in women, but not in men. Protein intake from either animal or vegetal sources was associated with total blood cholesterol levels, regardless of sex.

Our results are supported by prior studies that noted inverse associations between BP and protein from animal and vegetal food sources (16). The inverse association concerning meat-derived protein and DBP are unexpected, given that most studies reported positive associations between meat intake, either processed or unprocessed, and BP values (17, 18, 19, 20). However, beneficial effects of meat consumption on cardiometabolic health have also been reported (21). Wang et al. (22) noted that lean red meat consumption was associated with lower increases in BP in Chinese women compared with fatty meat intake. Wang et al. (23) examined more than 25,000 American women and reported that those who consumed <1.5 servings of red meat per day had a lower risk of hypertension. No associations were found between poultry consumption and hypertension risk. Golzarand et al. (24) reported similar findings. Results of a randomized clinical trial indicated that older women who adhered to a dietary pattern based on an average daily consumption of 85 g of lean red meat (e.g., beef, lamb, veal) had more pronounced reductions in BP than those who consumed ∼30 g/day (25). Hodgson et al. (26) found that replacing energy intake from carbohydrates with protein from lean red meat (∼43 g/day) reduced ambulatory and office BP, independent of age, sex, changes in body weight, alcohol intake, and urinary sodium or potassium excretion.

Possible explanations for these controversial findings lie in the associations between meat consumption with diets rich in saturated fatty acids (SFAs) and increased intake of heme iron (21). SFAs are a major risk factor for CVD by stimulating low-density lipoprotein (LDL) production (27). The content of SFAs in red meat is a major concern (28) and is the reason why this food is not included in healthy dietary patterns (e.g., Dietary Approaches to Stop Hypertension [DASH] diet) (25). Lean red meat and poultry have a low relative content of SFAs (28), which might explain their mild impact on CVD risk (25, 29, 30). Moreover, Bergeron et al. (31) reported that SFA content, rather “meat color”, has a pivotal role in atherogenesis.

Heme iron contributes to LDL oxidation through the generation of highly reactive hydroxyl-free radicals (32). Furthermore, evidence from large cohort studies indicates that dietary heme iron intake is positively associated with BP (33). Heme iron is mainly derived from animal sources (18, 32), and a higher content is found in red meat compared with chicken and pork (34).

Taken together, our results suggest that study participants might have consumed predominantly poultry and pork instead of fatty red meat. The resulting low ingestion of SFAs and heme iron might, in turn, have had beneficial effects on BP. It is also possible that a reduced red meat intake could be accompanied by a higher consumption of food items with hypotensive effects (e.g., fruits and vegetables) (25).

Significant negative associations were observed between blood glucose levels and protein sources from meat, meat derivates, fish, pasta, and bread. The beneficial effects of fish on glucose and insulin levels have repeatedly been reported (35). Fish oil consumption has shown to reduce fasting insulin and improve glucose tolerance in overweight individuals (36, 37). Such effects are likely mediated by an amelioration of intracellular insulin signaling in muscle and adipose tissue and a reduction of inflammation (38). The negative associations between protein from pasta or bread and cardiometabolic risk factors might be related to the consumption of fiber-rich flours/whole-grain foods (39, 40). The addition of specific fibers (e.g., viscous type) to pasta and bread lowers their glycemic index and reduces insulin secretion (39). Findings from an observational study indicated that the ingestion of whole grains was associated with increased insulin sensitivity and lower fasting insulin (40). Complementary results are provided by Kristensen et al. (41), who observed lower glucose concentrations over 90 min after the intake of whole-grain pasta and bread compared with the ingestion of the same foods prepared with refined flour.

The association between meat/meat derivates and blood glucose levels is actively debated. Although cohort studies have consistently suggested that red meat consumption might contribute to the development of type II diabetes mellitus (T2DM) (42, 43, 44), poultry consumption has been associated with a reduced risk of T2DM (45). Furthermore, Ibsen et al. (43) observed that the replacement of red meat with poultry significantly reduced the risk of T2DM in Danish individuals. A possible explanation is that poultry contains lower amounts of heme-iron than red meat (46). Talaei et al. (46) noted that the consumption of red meat and poultry was significantly associated with the incidence of T2DM after 10 years. However, only red meat remained significant when results were adjusted according to heme-iron intake. A complementary hypothesis is based on the action of branched-chain amino acids (BCAAs). Animal protein sources, mainly meat, are rich in BCAA (7), and some authors have raised the possibility that excess BCAA intake might cause a persistent activation of the S6K1/mTOR pathway, thereby promoting insulin resistance (47).

We also found significant and positive associations between total blood cholesterol and protein sources. This suggests that the simultaneous consumption of other macronutrients might have influenced our findings. Numerous traditional Italian recipes include high-fat sauces. For instance, popular pasta recipes, such as carbonara_3_ amatriciana, and alla gricia, include pork jowl and cheese (48), which are rich in SFAs (49) and can impact the formation of triacylglycerol by modulating the synthesis and activity of LDL receptors (50). Another example involves the consumption of bread and cereals, which are frequently eaten together with sweets or other fat-rich foods (e.g., cheese) (57). The positive associations observed for protein from meat, meat derivates, fish, milk, and yogurt might reflect a preference of study participants for high-fat products (e.g., whole milk, regular yogurt, sardine) rather than foods with lower fat contents.

Protein intake from a variety of food sources (e.g., bread, eggs, vegetables) was negatively associated with waist and hip circumferences and waist-to-hip ratio in both men and women. These results may have important clinical value, given that waist-to-hip ratio is an inexpensive and easy to apply index to estimate central adiposity. The latter is stronger associated with cardiovascular events, atherosclerotic disease, and diabetes than other anthropometric measures, such as BMI (44). A meta-analysis of observational studies that included more than 250,000 participants indicated that a 0.01 increase in the waist-to-hip ratio was associated with a 5% increase in the risk of a cardiovascular event (51).

A possible explanation to our findings is based on the effects of protein ingestion on satiety (52). Protein-rich diets are associated with greater satiety over the day, whereas low-protein intake is commonly associated with hunger before and after the main meals (53). Hunger is a major regulator of eating patterns (54). A greater appetite is associated with subsequent energy intake, involving the consumption of large amounts of food during discrete intervals, small amounts of food repeatedly over long periods, or snacks (54). Such a variation in meal patterns has an impact on hormonal homeostasis (55) and is associated with weight gain (56). In contrast, a high protein intake at breakfast and lunch stimulates the expression of genes that positively regulate lipid metabolism (57).

A last important finding of the present study is that protein intake from specific food groups was associated with cardiometabolic markers in either women or men. These results highlight the need for specific recommendations for both sexes and indicate that nutritional counseling is necessary to select protein sources that may promote muscle and cardiometabolic health.

Our study is not free of limitations. First, participants were mostly relatively young community-dwelling Caucasian older adults, and findings may not be applicable to other age or ethnic groups. Second, although participants were allowed to rest before BP measurement, it is possible that activities performed before the evaluation (e.g., walking, carrying bags) may have influenced the results. Third, blood glucose levels could have been affected by the participant fasting state. However, analyses were adjusted for self-reported fasting information. Fourth, only total blood cholesterol was measured, which precluded from evaluating associations between protein sources and cholesterol subfractions. Fifth, the FFQ used in the study included the weekly consumption of 12 food groups and was not validated in large samples sizes against standard methods (e.g., diet diary). This may explain the low energy and nutrient intake observed in our sample. Sixth, snacks, beverages, and alcohol were not included in the FFQ; therefore, they could not be accounted for in the regression analysis. Seventh, information on drugs for hypertension, diabetes, and dyslipidemia was collected through self-report and did not include drug classes or specific agents. Eighth, the presence of diseases was not recorded, which impeded to adjust our analyses according to hypertension, diabetes, dyslipidemia, metabolic syndrome, and other conditions. Ninth, the retrospective and cross-sectional design of the study does not allow inferring on temporal or cause-effect relationships between the variables considered.

## Conclusions

Findings of the present study indicate that several protein sources are favorably associated with BP and abdominal adiposity in both men and women. Inverse associations were observed between specific protein sources and blood glucose levels in women. Some protein sources showed positive associations with blood cholesterol in both sexes. These cross-sectional findings deserve further investigations using appropriate instruments and study designs.
